# Solution–Liquid–Solid Growth and Catalytic Applications of Silica Nanorod Arrays

**DOI:** 10.1002/advs.202000310

**Published:** 2020-05-27

**Authors:** Yaosi Fang, Kangxiao Lv, Zhao Li, Ning Kong, Shenghua Wang, Ao‐Bo Xu, Zhiyi Wu, Fengluan Jiang, Chaoran Li, Geoffrey A. Ozin, Le He

**Affiliations:** ^1^ Institute of Functional Nano & Soft Materials (FUNSOM) Jiangsu Key Laboratory for Carbon‐Based Functional Materials & Devices Soochow University 199 Ren'ai Road Suzhou Jiangsu 215123 P. R. China; ^2^ Solar Fuels Group Chemistry Department University of Toronto 80 St. George St Toronto Ontario M5S 3H6 Canada; ^3^ Department of Chemistry The University of Western Ontario London Ontario N6A 3K7 Canada

**Keywords:** catalysis, CO_2_ hydrogenation, nanorod arrays, silica nanostructures, solution–liquid–solid growth

## Abstract

As an analogue to the vapor–liquid–solid process, the solution–liquid–solid (SLS) method offers a mild solution‐phase route to colloidal 1D nanostructures with controlled sizes, compositions, and properties. However, direct growth of 1D nanostructure arrays through SLS processes remains in its infancy. Herein, this study shows that SLS processes are also suitable for the growth of nanorod arrays on the substrate. As a proof of concept, seedless growth of silica nanorod arrays on a variety of hydrophilic substrates such as pristine and oxide‐modified glass, metal sheets, Si wafers, and biaxially oriented polypropylene film are demonstrated. Also, the silica nanorod arrays can be used as a new platform for the fabrication of catalysts for photothermal CO_2_ hydrogenation and the reduction of 4‐nitrophenol reactions. This work offers some fundamental insight into the SLS growth process and opens a new avenue for the mild preparation of functional 1D nanostructure arrays for various applications.

## Graphical abstract

Seedless solution–liquid–solid growth of silica nanorod arrays on a variety of hydrophilic substrates is demonstrated. These nanorod arrays provide a new versatile platform for the fabrication of supported metal catalysts for various purposes.

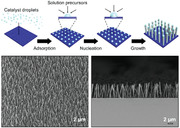

## 1 Introduction

The vapor–liquid–solid (VLS) method has been widely used in the preparation of high‐quality 1D nanostructures with a broad range of compositions.^[^ ^1^ ^]^ Owing to their unique properties, such as short charge diffusion distances, light trapping ability, and ease of device integration, VLS grown nanowire/nanorod arrays have attracted particular attention.^[^ ^2^ ^]^ For example, Wang et al. demonstrated the VLS growth of ZnO nanowire arrays and their applications in piezoelectric nanogenerators.^[^ ^3^ ^]^ Cui et al. found that nanowire arrays are promising structures for Li‐ion batteries due to their facile strain relaxation, short Li^+^ diffusion distances, and good electronic conduction.^[^ ^4^ ^]^ Yang et al. reported the integration of silicon nanowire arrays with bacteria as a catalyst for photoelectrochemical reduction of CO_2_ to value‐added chemicals.^[^ ^5^ ^]^ Atwater and his co‐workers have demonstrated that high‐quality Si wire arrays exhibit advantageous optical properties for photovoltaic applications.^[^ ^6^ ^]^

As an analogue to VLS methods, solution–liquid–solid (SLS) growth has emerged as an alternative approach for preparing 1D nanostructures, including groups IV, II–VI, III–V, and IV‐VI compounds.^[^ ^7^ ^]^ In a typical SLS growth process, the catalyst droplets (liquid phase) are dispersed in a solvent (solution phase) to form an emulsion system. The precursor diffuses from the solution phase into the liquid phase to initiate the chemical reaction for producing monomers. When monomer concentration increases to a critical point, nucleation and growth of 1D nanostructures (solid phase) is then triggered. Compared to the VLS route, solution‐phase SLS processes feature milder growth conditions with temperatures usually below 350 °C and produce colloidal 1D nanostructures with narrow size distributions, good dispersity, and ease of post‐functionalization.^[^ ^8^ ^]^ Recently, several studies have shown that, in the presence of colloidal seeds onto which the catalyst droplet is adsorbed, 1D nanostructures could also grow from the seed surface for the preparation of complex heterostructured particles.^[^ ^9^ ^]^ However, to the best of our knowledge, there are very few reports on the seedless growth of 1D nanostructure arrays via the SLS mechanism, hindering the practical applications of SLS methods.

In this study, we show that SLS processes are ideal enablers for the growth of nanorod arrays on the substrate. As a proof of concept, we demonstrate seedless growth of silica nanorod arrays on different hydrophilic substrates. The key of the seedless growth process is the adsorption of catalyst droplets onto the substrate with suitable surface wettability. By controlling the growth conditions, nanorod arrays with controlled structure parameters can be obtained. We further demonstrate the use of silica nanorod arrays as a new platform for the fabrication of supported catalysts with enhanced performance in photothermal hydrogenation of carbon dioxide. Moreover, SiO_2_ arrays decorated with Au nanoparticles show good activity in the catalytic reduction of 4‐nitrophenol.

## 2 Results and Discussions

### 2.1 Growth Mechanism

**Figure 1:**
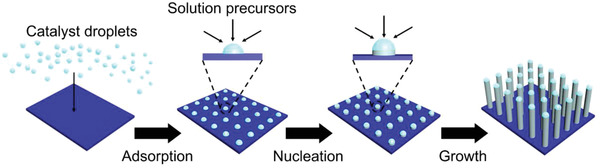
Schematic illustration of the seedless growth of silica nanorod arrays on the substrate.

**Figure** **1** illustrates the SLS process for seedless growth of silica nanorod arrays on the substrate. In the first step, the mixing of an aqueous sodium citrate solution containing ammonium hydroxide with 1‐pentanol produces a water‐in‐alkanol emulsion due to the poor solubility of sodium citrate in 1‐pentanol. In the second step, the adsorption of aqueous catalyst droplets onto the hydrophilic substrate is driven by the reduction of the surface energy. Specifically, the surface energy change (Δ*E*) for the adsorption of a catalyst droplet with a radius of *R*_0_ into the flat substrate can be described by the following equation.

(1) $$\Delta E=\gamma_{12}\pi R_{0}^{2}\left(\sqrt[{3}]{16\;\left(2-3\cos\theta +\cos^{3}\theta \right)}-4\right)$$

where *γ*_12_ is the surface tension at the solution‐liquid interface (between the pentanol phase and the aqueous droplet) and *θ* is the contact angle of the catalyst droplet on the substrate surface. As this angle decreases, the absolute value of Δ*E* becomes bigger, that makes adsorption on hydrophilic substrate (*θ* < 90°) thermodynamically favorable (see Supporting Information for detailed calculations). Moreover, the adsorbed droplet is more stable for substrates with higher hydrophilicity, which is the key for the seedless growth of silica nanorod arrays. Next, the third step involves the addition of tetraethyl orthosilicate (TEOS) into the reaction to trigger the nucleation and growth of silica nanorods from the adsorbed droplets. Finally, in the fourth step silica nanorod arrays are obtained after removing the substrate from the growth solution.

### 2.2 Growth of Silica Nanorod Arrays on ITO Glass

**Figure 2:**
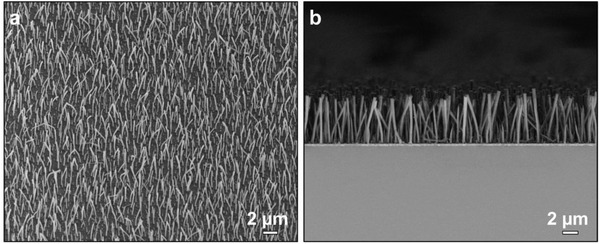
SEM images of a typical sample of SiO_2_ nanorod arrays grown on the ITO glass slide viewed from different directions. The growth temperature was 60 °C and the growth time was 4 h.

Experimentally, we first demonstrated the growth of silica nanorod arrays from the ITO glass slide. **Figure** **2**a shows the scanning electron microscopy (SEM) image of typical silica nanorod arrays grown from ITO glass. A forest of silica nanorods was found to grow vertically from the ITO substrate. The cross‐section SEM image revealed that the silica nanorods were relative uniform in diameter and length (Figure 2b). Statistics from 40 nanorods showed that the average length and diameter of the as‐obtained nanorods were 4.54 ± 0.44 and 0.28 ± 0.03 µm, respectively. Notably, these sol–gel prepared silica nanorods were amorphous and thus exhibited a certain degree of flexibility; this accounts for the slightly bent nanorods as observed in the SEM images (Figure 2a,b).^[^ ^10^ ^]^ The capillary/Laplace forces of the moveable catalyst droplets may also cause the nanorod bending toward another.^[^ ^11^ ^]^ These results reinforce the effectiveness of our strategy in the seedless growth of silica nanorod arrays from the ITO substrate.

### 2.3 Growth Control

**Figure 3:**
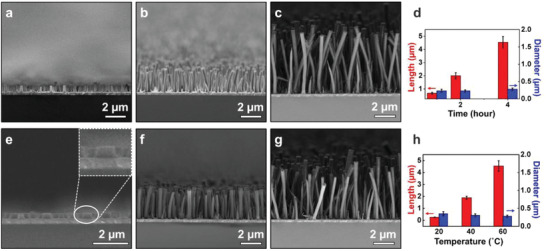
SEM images of SiO_2_ nanorod arrays grown on ITO glass substrates obtained at different growth times: a) 1, b) 2, and c) 4 h. d) The dependence of nanorod length and diameter on the growth time; SEM images of SiO_2_ nanorod arrays grown on ITO glass substrates at different temperatures: e) 20, f) 40, and g) 60 °C. h) The dependence of nanorod length and diameter on the growth temperature.

Based on the SLS growth mechanism, the diameter and length of silica nanorods could be conveniently tuned by controlling the reaction conditions. **Figure** **3**a–c depict the SEM images of silica nanorod arrays at different growth times. The length of nanorods increased from 0.68 ± 0.07 to 2.01 ± 0.22 µm when the reaction time was lengthened from 1 to 2 h (Figure 3d). Further increase of the growth time to 4 h led to the formation of nanorods with an average length of 4.54 ± 0.44 µm. The diameter of nanorods barely changed among these three samples, which is consistent with the 1D growth mechanism of SLS processes. Moreover, the growth rate of SiO_2_ nanorods could be controlled by changing the reaction temperature, providing additional opportunities for tailoring their structural parameters. A series of silica nanorod arrays were grown at different temperatures ranging from 20 to 60 °C while keeping the reaction time fixed, and their SEM images are depicted in Figure 3e–g. The nanorod length increased from 0.26 ± 0.02 to 1.89 ± 0.13 µm when the reaction temperature was raised from 20 to 40 °C (Figure 3h), and a further increase to 60 °C led to the formation of nanorods with an average length of 4.54 ± 0.44 µm. The nanorod diameter decreased from 0.35 ± 0.06 to 0.31 ± 0.04 to 0.28 ± 0.03 µm for these three samples. These results indicate the growth temperature could affect both the growth rate of silica nanorods and the amount of water in the catalyst droplets, which allows the shape of silica nanorod arrays to be adjusted without changing the reaction solution.

In addition, we performed experiments to verify the effectiveness of manipulating the reaction temperature to achieve control of the spatial diameter of each silica nanorod. More complex structures can be prepared by repeatedly shifting the reaction temperature from 60 to 20 °C. We observed that structural parameters correlate with temperature in Figure S1, Supporting Information: higher temperature lead to smaller diameters and faster growth rates, and this is reflected by the length and diameter of silica nanorods in Figure S1a, Supporting Information. Figure S1b, Supporting Information exhibits the SEM images of spindle‐shaped silica arrays with 380 nm diameter in the middle and 300 nm diameter at both sides. This is because at a given point in time, the local diameter of silica nanorods depends on the catalyst droplet size. At hotter temperature, the increased solubility of water in alkanol causes the droplet to shrink and accelerates the growth rate of silica nanorods. Given that nanorods of various lengths, thicknesses, and with non‐uniform diameters can be synthesized, this SLS route provides a convenient way of controlling the morphology of nanorod arrays, greatly increasing the diversity of nanorod arrays via liquid‐phase synthesis.

### 2.4 Versatility of the Growth Method

**Figure 4:**
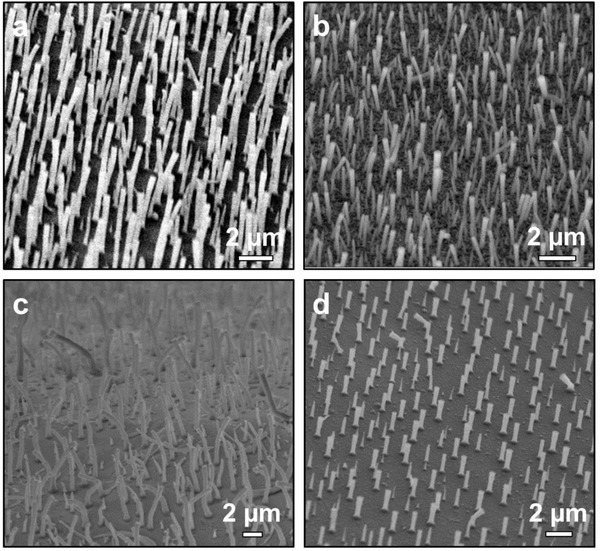
SEM images of SiO_2_ nanorods grown on different substrates: a) pristine glass, b) FTO glass, c) titanium sheets, and d) plasma‐treated Si wafers.

We now demonstrate the versatility of the seedless SLS strategy by extending it to the growth of silica nanorod arrays on other substrates, namely pristine glass, FTO glass, titanium sheets and silicon wafers (**Figure** **4**). All substrates other than the Si wafers are hydrophilic, as indicated by their low contact angles with water (Figure S2, Supporting Information). Similar to ITO glass, silica nanorod arrays could be directly grown from pristine glass, FTO substrates, and titanium sheets (Figure 4a–c). Better uniformity in the orientation of silica nanorods was observed for pristine glass in Figure S3, Supporting Information, suggesting that nanorod growth was affected by the smoothness of the substrate. Interestingly, the morphology of silica nanorod arrays can also be manipulated on pristine glass by controlling the growth time and temperature (Figure S4, Supporting Information). For silicon wafers with less hydrophilicity, growth of silica nanorod arrays was achieved only after plasma pre‐treatment to increase the substrate hydrophilicity (Figure 4d; Figures S5 and S6, Supporting Information). Thanks to the low reaction temperature, our strategy can be also extended to grow silica nanorod arrays on hydrophilic flexible polymer substrates (Figure S7, Supporting Information). These results clearly demonstrate the effectiveness of our strategy in the growth of silica nanorod arrays on the substrate with suitable wettability. Furthermore, our strategy is potentially suitable for the site‐selective growth of silica nanorods from substrates with spatially patterned wettability.

As shown above, our strategy is effective for seedless growth of silica nanorod arrays on different substrates. By taking advantages of the well‐developed silane chemistry, the surface of silica nanorods could easily be modified with various functional groups. As shown in Figure S8, Supporting Information, after modification with fluoro silane, the surface of silica nanorod arrays can be switched from hydrophilic to hydrophobic with the increase of contact angle from 15.2˚ to 139.6˚. In contrast, the contact angle of bare ITO glass only increased by 39˚. This result indicated that SiO_2_ is superior to other metal oxides on the surface post‐treatment via silane chemistry.^[^ ^12^ ^]^ Additionally, the micro‐nano structure of SiO_2_ nanorod arrays provides a further increase of hydrophobicity since the contact angle of pristine glass is 110˚ in a similar surface modification procedure.^[^ ^13^ ^]^ Moreover, the as‐obtained silica nanorods could serve as seeds for the growth of secondary materials to produce 3D hierarchical nanostructures. As an example, we demonstrate the deposition of a nickel oxyhydroxide (NiOOH) layer on the surface of silica nanorods. Figure S9, Supporting Information depicts the SEM images of the as‐obtained composite nanorod arrays, denoted as NiOOH@SiO_2_. Each silica nanorod was conformably coated with a layer of flower‐like NiOOH, forming a core‐shell structure. Furthermore, after reduction in a hydrogen atmosphere, Ni@SiO_2_ nanorod arrays can be obtained as shown in Figure S10, Supporting Information. The growth of secondary nanostructures from the silica nanorods provides an effective way to expand the family of nanorod arrays with different compositions and functionalities.

### 2.5 Applications in Catalysis

**Figure 5:**
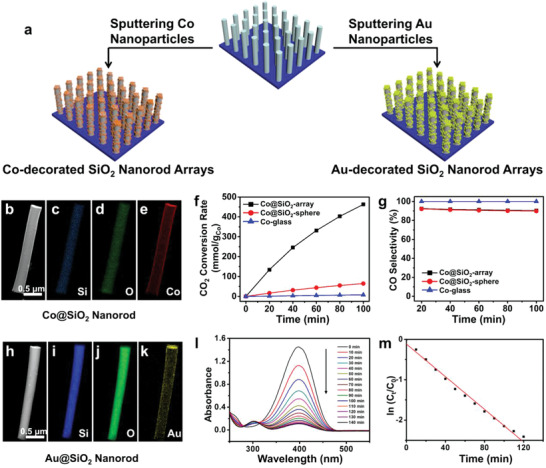
a) Schematic illustration of the sputtering different metals onto the surface of SiO_2_ nanorod arrays. b–e) Element mapping of one rod from the Co@SiO_2_‐array sample. f–g) Photothermal catalytic performance of Co@SiO_2_‐array (in black), Co@SiO_2_‐sphere (in red), and Co‐glass (in blue). h–k) Element mapping of one rod from the Au@SiO_2_‐array sample. l) Time‐dependent UV−vis spectra of the reaction solution in the presence of Au@SiO_2_‐array for 10‐min intervals. m) Plot of ln (*C*_t_/*C*_0_) versus the duration of the reduction reaction. *C*_t_ and *C*_0_ are the concentrations of 4‐nitrophenol at time *t* and time 0, respectively.

We further demonstrate the use of the silica nanorod arrays as general support materials for various catalytic applications through the functionalization by, but not limited to, physical methods. For example, the as‐obtained silica arrays could serve as a universal platform for functionalization with metal nanoparticles to form metal‐silica composites, denoted as M@SiO_2_‐array (**Figure** **5**a). In this study, Co@SiO_2_‐array and Au@SiO_2_‐array nanostructures were prepared by sputtering, and used in gas‐phase photothermal catalysis and liquid‐phase catalysis, respectively (Figure 5b–e,h–k).^[^ ^14^ ^]^

The activity of Co@SiO_2_‐array in photothermal CO_2_ catalysis was tested in a batch reactor under 2.4 W cm^−2^ light intensity without external heating. Three control samples were also used, including SiO_2_ nanorod arrays without Co (denoted as SiO_2_‐array), Co nanoparticles on the pristine glass (denoted as Co‐glass), and Co nanoparticles supported on silica spheres (denoted as Co@SiO_2_‐sphere, Figure S11, Supporting Information). The amount of Co measured by the inductively coupled plasma source mass (ICP‐MS) was found to be 0.176 mg for Co‐glass, Co@SiO_2_‐array, and Co@SiO_2_‐sphere. The control sample of SiO_2_‐array was inert to the CO_2_ conversion. As shown in Figure 5f, the CO_2_ conversion rate of Co@SiO_2_‐array reached 407.3 mmol g_Co_^−1^ h^−1^ (normalized by the mass of Co) in the first 20 min of reaction, which is eight times and 140 times as high as that of Co@SiO_2_‐sphere and Co‐glass, respectively. This may be due to differences in light utilization, as multiple reflections and wave‐guiding in the nanoarray significantly enhanced the light pathway (Figure S12, Supporting Information).^[^ ^15^ ^]^ These samples gave similar CO selectivity of more than 90% as shown in Figure 5g. The higher selectivity of Co‐glass is because the yield of CH_4_ was below the detection limit of the instrument. These results clearly demonstrate the advantage of silica nanorod arrays as the catalyst support for photothermal CO_2_ catalysis.

We also investigated the catalytic performance of Au@SiO_2_‐array in the reduction of 4‐nitrophenol to 4‐aminophenol (Figure 5l) by using bare glass with same amount of Au nanoparticles as a control.^[^ ^16^ ^]^ The catalytic result shows that the activity of Au@SiO_2_‐array is seven times higher than that without arrays (Figure 5m; Figure S13, Supporting Information). It is worth noting that the experiments were carried out without stirring. Benefiting from the advantages of arrays and hydrophilic SiO_2_, Au@SiO_2_‐array present a comparable conversion rate per unit mass to the nanocomposite powders (Table S1, Supporting Information). Moreover, the recycling of Au@SiO_2_‐array after the catalytic testing is very convenient. These results suggested silica nanorod arrays is a promising platform in serving as catalyst support for their advantages: the light trapping effect, a large surface area, compactness, ease of handling and recycling, and facilitation of gas/liquid molecular transport.

## 3 Conclusion

In summary, we have demonstrated controlled SLS growth of silica nanorod arrays on various hydrophilic substrates. The as‐obtained nanorod arrays provide a new platform for the fabrication of supported metal catalysts for various purposes, such as photothermal CO_2_ hydrogenation and the reduction of 4‐nitrophenol. Our strategy is potentially suitable for the site‐selective growth of silica nanorods from substrates with spatially patterned wettability. Furthermore, the surface of silica nanorods could easily be modified with various functional groups through the well‐established silane chemistry to find applications in many other areas. Our work reveals the potential of SLS processes as mild solution‐phase methods for the direct growth of 1D nanostructure arrays. By choosing suitably designed substrates, this strategy could be extended to grow 1D nanostructure arrays of more functional materials, such as TiO_2_ (Figure S14, Supporting Information), Si, Ge, GaAs, InP, InAs, InN, CdSe, ZnSe, GaP, and PbS‐PbSe.^[^ ^7^, ^8^ ^]^ Thanks to its relatively mild growth conditions, the SLS method might be more compatible for the scalable growth of functional nanoarrays on flexible substrates with poor thermal stability toward the applications in flexible electronics.

## 4 Experimental Section

##### Materials

Si (111) wafer (phosphorus‐doped), ITO‐coated glass (ITO thickness = 150–300 Å), FTO‐coated glass (FTO thickness = 150–300 Å), polyvinylpyrrolidone (PVP, *M*_n_ = 55 000), 1‐pentanol and 1H,1H,2H,2H‐perfluorooctyl trichlorosilane [CF_3_(CF_2_)_5_(CH_2_)_2_SiCl_3_, PFTS] were purchased from Aldrich. Sodium citrate (C_6_H_5_NaO_7_, >98%) was purchased from Energy Chemical (Shanghai). Ammonium hydroxide (NH_3_·H_2_O, 25–28% NH_3_ in water) was obtained from Mackin Biochemical (Shanghai) co., Ltd. Tetraethyl orthosilicate (TEOS, 96%) was purchased from TCl (Shanghai) development co., Ltd. Milli‐Q water (Millipore, 18.2 MΩ cm at 25 °C) was used in all experiments.

##### Pretreatment of Substrates

ITO‐coated, FTO‐coated, and pristine glass slides, were successively cleaned using acetone, water, and ethanol prior to use. For Si wafers, an additional treatment with oxygen plasma for 30 min was employed to improve the substrate hydrophilicity.

##### Synthesis of Silica Nanorod Arrays

In a typical experiment, 1 g of PVP was dissolved in 10 mL of 1‐pentanol by sonication, followed by the addition of 380 µL of aqueous sodium citrate solution (0.053 m). The pre‐treated substrate was then immersed in the above solution. 200 µL of ammonium hydroxide solution and 1 mL of ethanol were added into the reaction. Strong agitation was provided by sonication to form a stable emulsion system. 100 µL of TOES was slowly added to the reaction under sonication during a period of 20 s. The mixed solution was kept standing quiescently for a certain period of time at different temperatures. Finally, the substrate was removed from the solution, rinsed several times with water and ethanol, and dried in a vacuum oven.

##### Hydrophobic Modification

The surface modification was monitored by grafting PFTS onto the surfaces. 50 µL of PFTS was dropped to a piece of Wipes at the bottom of a vacuum desiccator. Silica arrays and different substrates were paced face up in the same vacuum desiccator. The set was vacuumed for 10 min, standing still for half an hour. Then the samples were taken out from the vacuum desiccator and wash with water for several times.

##### Growth of NiOOH on Silica Nanorod Arrays

In a typical synthesis of NiOOH@SiO_2_ arrays, the as‐obtained SiO_2_ nanorod arrays were immersed in a mixture of 10 mL of 1 m nickel sulfate and 7.5 mL of 0.25 m potassium. After adding 2.5 mL of ammonia, the reaction mixture was kept standing at room temperature for 20 min. Then the substrate was taken out from solution and washed several times with distilled water. The sample could be further converted to NiO@SiO_2_ or Ni@SiO_2_ arrays by calcination in air or hydrogen atmosphere at 300 °C.

##### Preparation of Metal‐Decorated SiO_2_ Nanorod Arrays by Magnetron Sputtering

The metal deposition was carried out in a custom‐built sputtering system (Kurt J. Lesker Co.) by radio frequency magnetron sputtering using a 99.95% pure Co or Au sputtering target purchased from Zhongnuo Advanced Material Co., Beijing China. The base pressure of the sputtering chamber was pumped down to 1×10^−5 ^Torr before argon was introduced into the chamber at a flow rate of 86 sccm. The chamber pressure was set to 9.8×10^−3 ^Torr during the deposition, which was carried out at room temperature. The distance between substrate and target was 17.5 cm with the forward power of 100 W. During the preparation of these samples, the amount of metal was controlled by time of sputtering and the accurate amount of metal was measured by ICP‐MS.

##### Preparation of Co@SiO_2_‐Sphere and Co‐Glass

These two samples were also prepared by magnetron sputtering under the same conditions as Co@SiO_2_‐array. For Co@SiO_2_‐sphere, an aqueous dispersion of 600‐nm SiO_2_ spheres (50 mg) was drop‐casted onto the glass substrate and dried naturally to form a thin film. The amount of Co measured by ICP‐MS was found to be the same for Co‐glass, Co@SiO_2_‐array, and Co@SiO_2_‐sphere.

##### Photocatalytic CO_2_ Hydrogenation

The gas‐phase photocatalytic experiments were conducted in a batch reactor with internal gas circulation. The pressure inside the reactor was monitored during the reaction using an MIK‐P300 pressure transducer. A 300‐W Xe arc lamp was used to illuminate the catalysts without any filter. The reactor was degassed and purged with a mixture of CO_2_ and H_2_ (1:1) twice. The reactor was then sealed when the pressure inside reached 1 bar, and the light was turned on to initiate the photocatalytic reaction. The amounts of gas products (CO and CH_4_) were analyzed with a flame ionization detector installed in a GC‐7900 gas chromatograph (GC) (TECHCOMP).

##### Catalytic Reduction of 4‐Nitrophenol

A certain amount of Au nanoparticles was sputtered on the silica nanorod arrays on a glass slide. Afterward, the sample was cut into small squares no larger than 1 cm × 1 cm. For the catalytic reduction of 4‐nitrophenol, 1 mL of aqueous solutions of 4‐nitrophenol (2×10^−5^ m) and 1 mL of freshly prepared aqueous NaBH_4_ solution (0.1 m) were added into a quartz cuvette. After adding the catalysts, the bright yellow solution gradually faded as the catalytic reaction proceeded. The UV–vis absorption spectra of the solution were monitored during the reaction. Control experiments were carried out under similar conditions using pristine glass slide without silica nanorod arrays.

##### Characterizations

SEM images were collected using a Gemini‐500 Zeiss SEM. TEM images were obtained using a F20 FEI TEM with an acceleration voltage of 200 kV. The contents of Au and Co were measured by an ICP‐MS (Aurora M90, Jenoptik). Contact angles on arrays and substrates were measured using deionized water with a contact angle meter (Dataphysics Instrument, Dataphysics OCA) in an ambient atmosphere at room temperature. The UV–vis spectra were measured using a probe type Ocean Optics QE65 Pro UV–vis spectrophotometer in absorption mode with an integration time of 8 ms.

## Conflict of Interest

The authors declare no conflict of interest.

## Supporting information

Supporting Information
