# Supplementary material for: Solution–Liquid–Solid Growth and Catalytic Applications of Silica Nanorod Arrays
Source: Adv Sci (Weinh). 2020 May 27;7(13):2000310. doi: 10.1002/advs.202000310 (PMC7341079; doi:10.1002/advs.202000310)
Supplement: Supplementary file 1 — Supporting Information [file ADVS-7-2000310-s001.pdf]

((Supporting Information can be included here using this template))

Copyright WILEY-VCH Verlag GmbH & Co. KGaA, 69469 Weinheim, Germany, 2018.

Supporting Information

Solution-Liquid-Solid Growth and Catalytic Applications of Silica Nanorod Arrays

Yaosi Fang, Kangxiao Lv, Zhao Li, Ning Kong, Shenghua Wang, Ao-Bo Xu, Zhiyi Wu, Fengluan Jiang, Chaoran Li, Geoffrey A. Ozin* and Le He**

Calculation of the energy change of adsorption on substrate

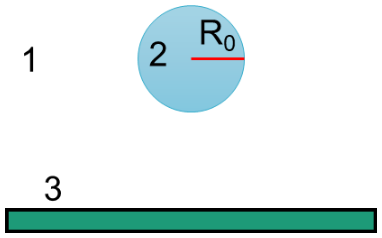

Considering an emulsion droplet in pentanol phase with radius of R_0 and volume of $\frac{4}{3}\pi R_0^3$. In the absence of gravity, the total energy of the free droplet state (before adsorption) can be expressed by the surface energy of the three phases (1 denotes the pentanol phase, 2 denotes the droplet phase, and 3 denotes the wafer substrate).

$$E = \gamma_{12}S_{12} + \gamma_{13}S_{13} \quad (1)$$

where γ_{12} is the surface tension at the solution-liquid interface (between the pentanol phase and the droplet), S_{12} is the contact area between the pentanol phase and the catalyst droplet, γ_{13} is the surface tension at the solution-solid interface (between the pentanol phase and substrate surface), and S_{13} is the contact area between the pentanol phase and the substrate.

The energy change (ΔE) of the adsorption process can be calculated as:

$$\Delta E = \gamma_{12}\Delta S_{12} + S_{23}(\gamma_{23} - \gamma_{13}) \quad (2)$$

where S_{23} is the contact area of the liquid-solid interface (between the droplet and the substrate surface) after the adsorption and γ_{23} is the surface tension at the liquid-solid interface. Note $S_{23} = -\Delta S_{13}$.

Now we calculate the hydrophilic dependence of the energy change on the smooth flat surface.

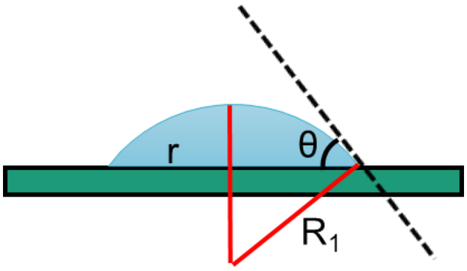

At equilibrium state, the Young–Dupré equation must be satisfied

$$\gamma_{13} - \gamma_{23} = \gamma_{12} \cos\theta \quad (3)$$

Substitute equation (3) into equation (2) gives

$$\Delta E = \gamma_{12}(\Delta S_{12} - \cos\theta\, S_{23}) \quad (4)$$

The change of contact area between the catalyst droplet and the pentanol phase is

$$\Delta S_{12} = 2\pi R_1^2(1 - \cos\theta) - 4\pi R_0^2 \quad (5)$$

where R_1 is the radius of curvature of the droplet.

The contact area between the catalyst droplet and the substrate surface is

$$S_{23} = \pi R_1^2 \sin^2\theta \quad (6)$$

The conservation of the droplet volume gives

$$\frac{4}{3}\pi R_0^3 = \frac{1}{3}\pi R_1^3(2 - 3\cos\theta + \cos^3\theta) \quad (7)$$

Equation (7) can be written as

$$R_1 = \sqrt[3]{\frac{4}{(2-3\cos\theta+\cos^3\theta)}}\, R_0 \quad (8)$$

Substitute equation (8) into equation (5) gives

$$\Delta S_{12} = \pi R_0^2 \left(\sqrt[3]{\frac{128\,(1-\cos\theta)^3}{(2-3\cos\theta+\cos^3\theta)^2}} - 4\right) \quad (9)$$

Substitute equation (8) into equation (6) gives

$$S_{23} = \pi R_0^2\,(1 - \cos^2\theta)\,\sqrt[3]{\frac{16}{(2-3\cos\theta+\cos^3\theta)^2}} \quad (10)$$

Substitute equations (9) and (10) into equation (4) gives

$$\Delta E = \gamma_{12}\pi R_0^2\left(\sqrt[3]{\frac{128\,(1-\cos\theta)^3}{(2-3\cos\theta+\cos^3\theta)^2}} - 4 - \cos\theta\,(1-\cos^2\theta)\sqrt[3]{\frac{16}{(2-3\cos\theta+\cos^3\theta)^2}}\right)$$

$$= \gamma_{12}\pi R_0^2\left(\sqrt[3]{16\,(2-3\cos\theta+\cos^3\theta)} - 4\right) \quad (11)$$

The following figure shows the contact angle dependence of energy change.

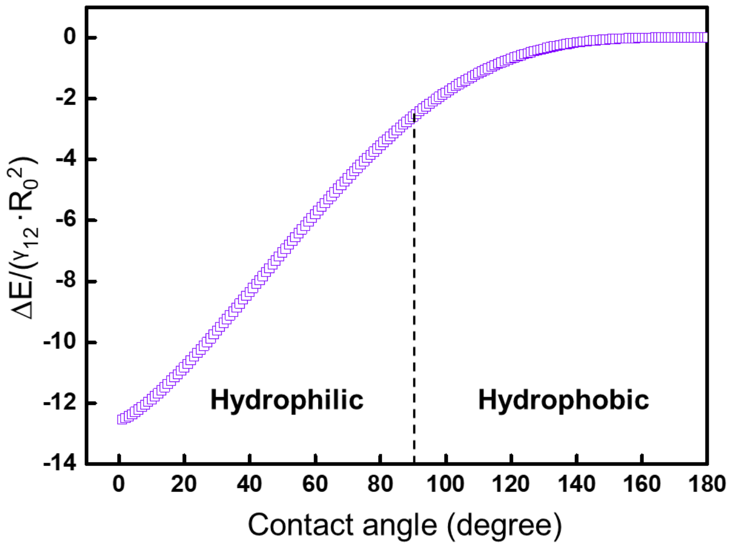

Supporting Figures

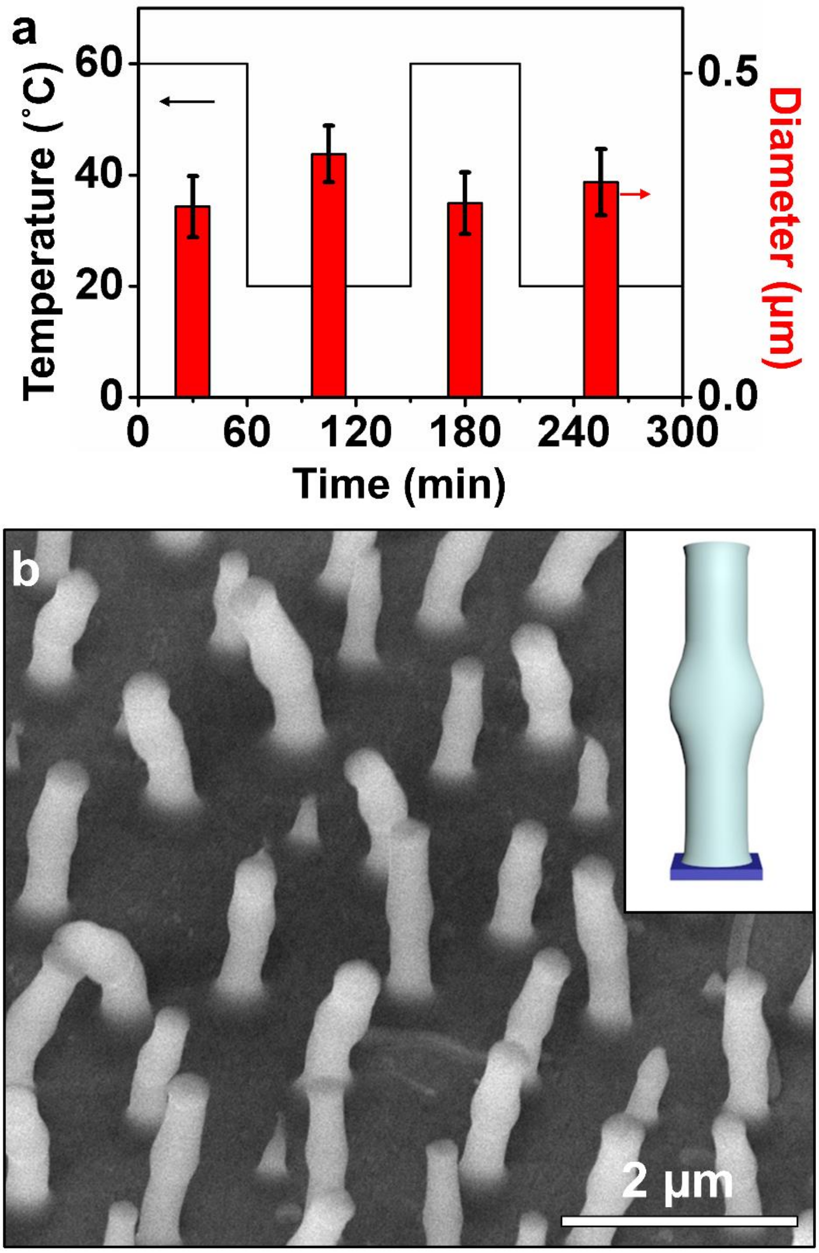

Figure S1. a) The plot of growth conditions and the nanorod diameter at various points in time. **b**) SEM images of spindle-shaped SiO_2 nanorod arrays grown on ITO glass substrates.

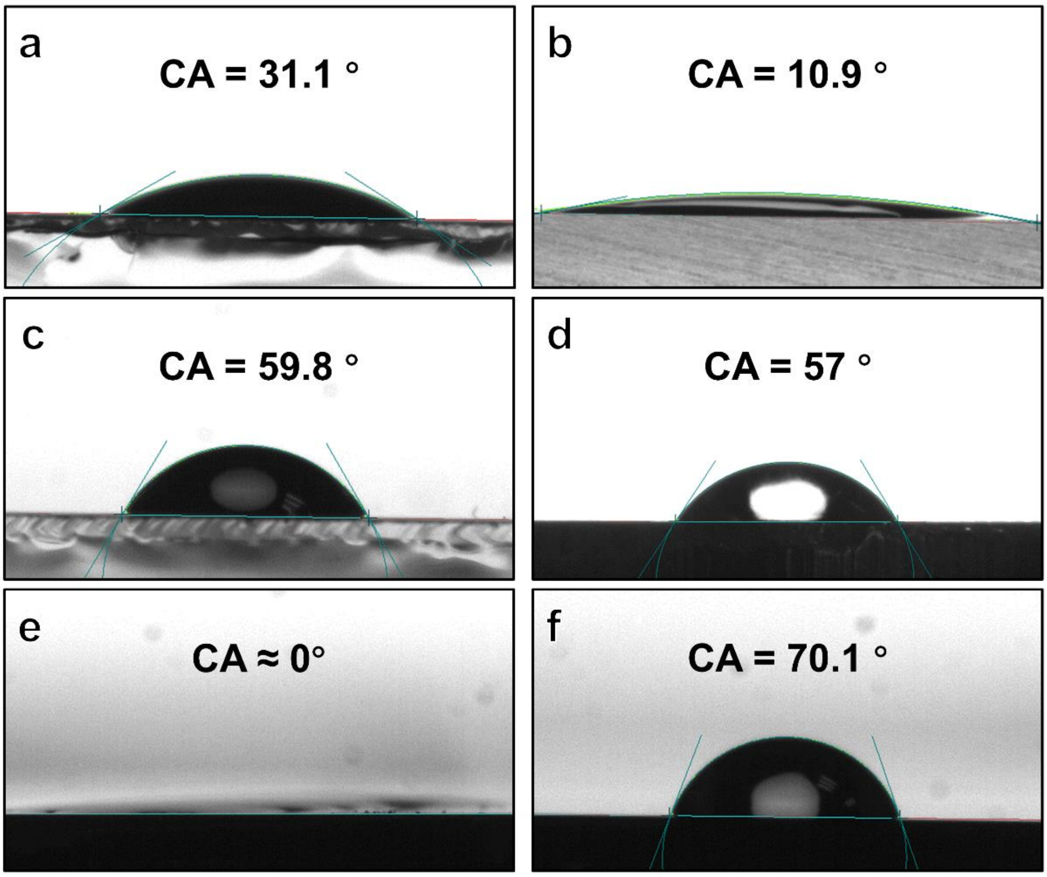

Figure S2. The contact angles of water on these substrates. **a**) ITO glass, **b**) pristine glass, **c**) FTO glass, **d**) titanium sheets, **e**) plasma-treated Si wafer and **f**) untreated Si wafer.

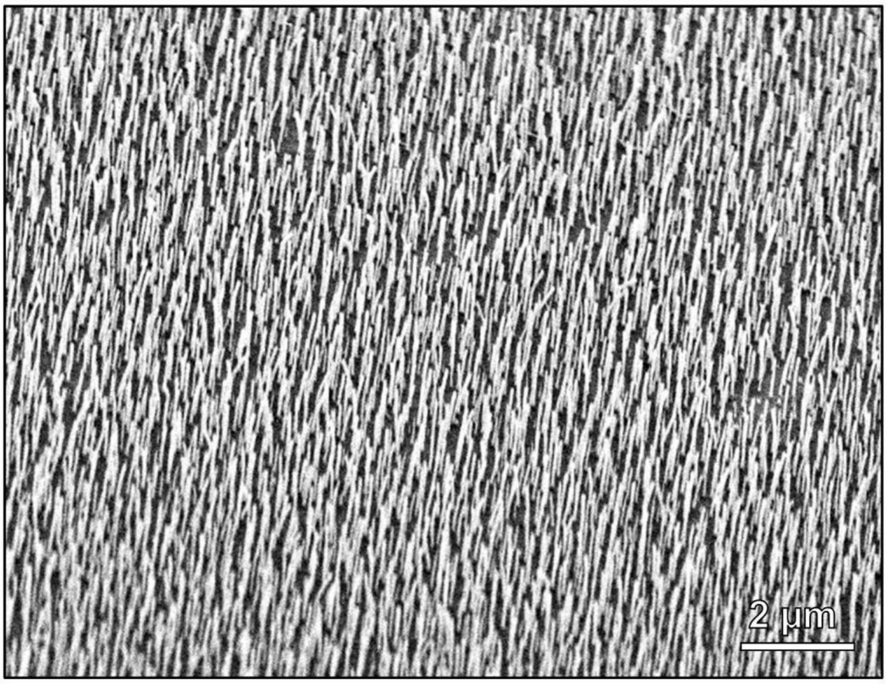

Figure S3. SEM image of silica nanorod arrays grown on pristine glass.

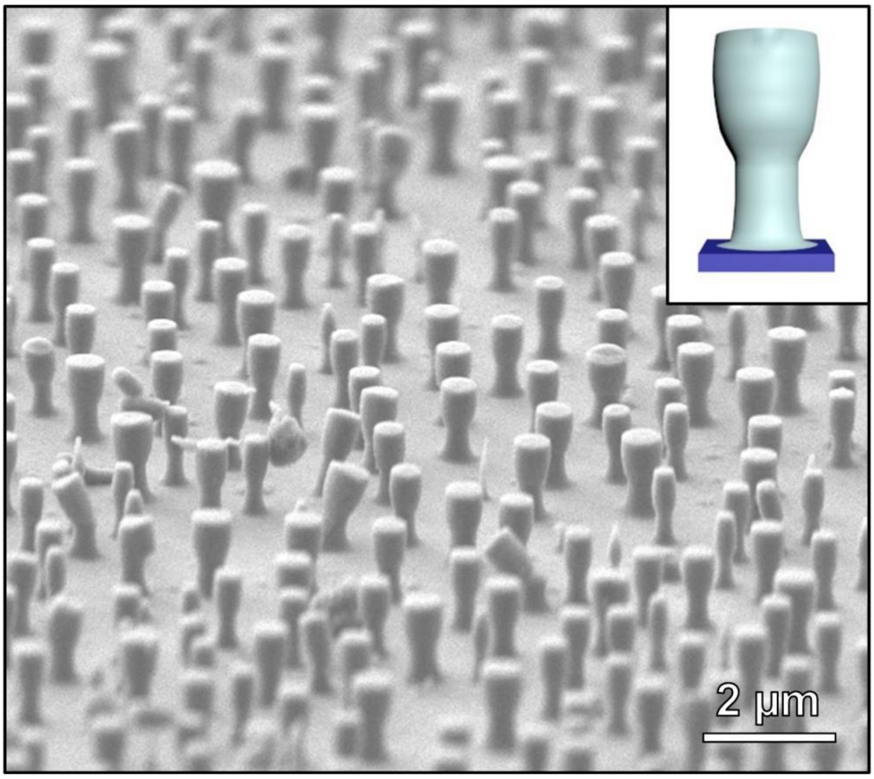

Figure S4. SEM image of goblet-like silica arrays. This was obtained by growing at 60 ℃ for 30 minutes then cooling gradually (1°C/min) to 20 ℃ for another 2 hours.

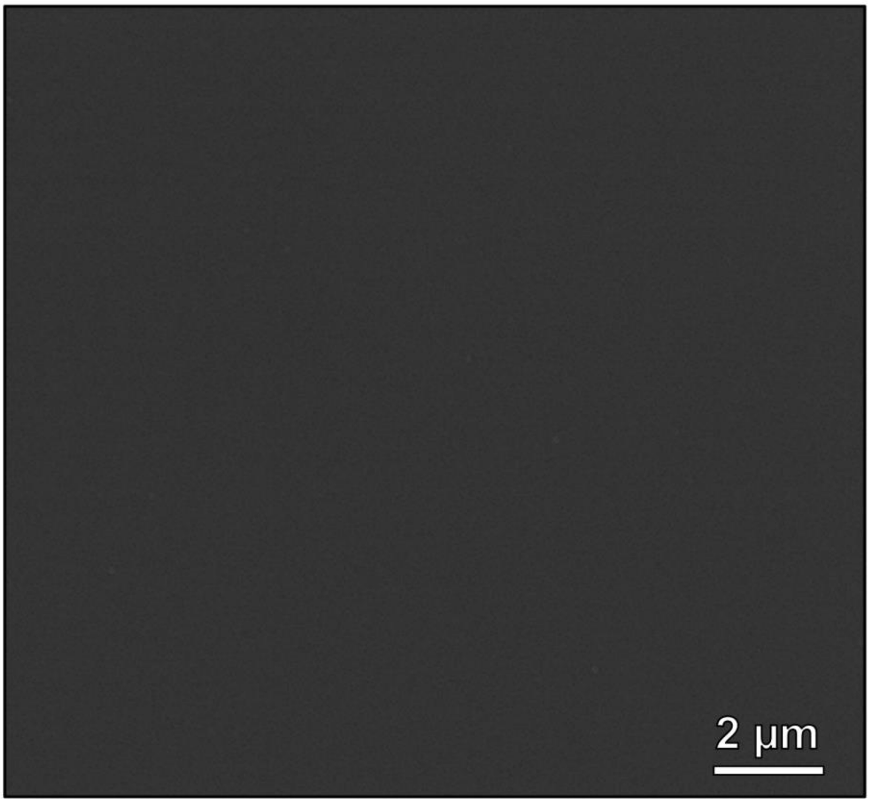

Figure S5. SEM image of the growth situation through the same process as seen in Figure 4d using silicon wafer without plasma treatment. None silica nanorods were found on the substrate.

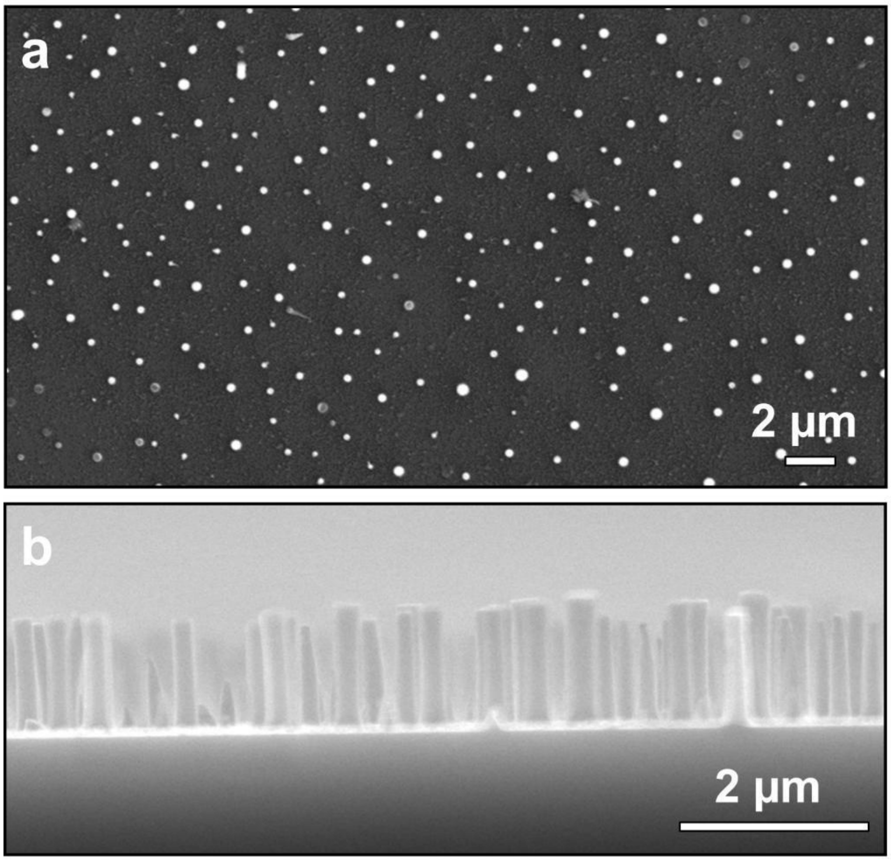

Figure S6. SEM images of the silica nanorod arrays in Figure 4d viewed from the **a**) top and **b**) side.

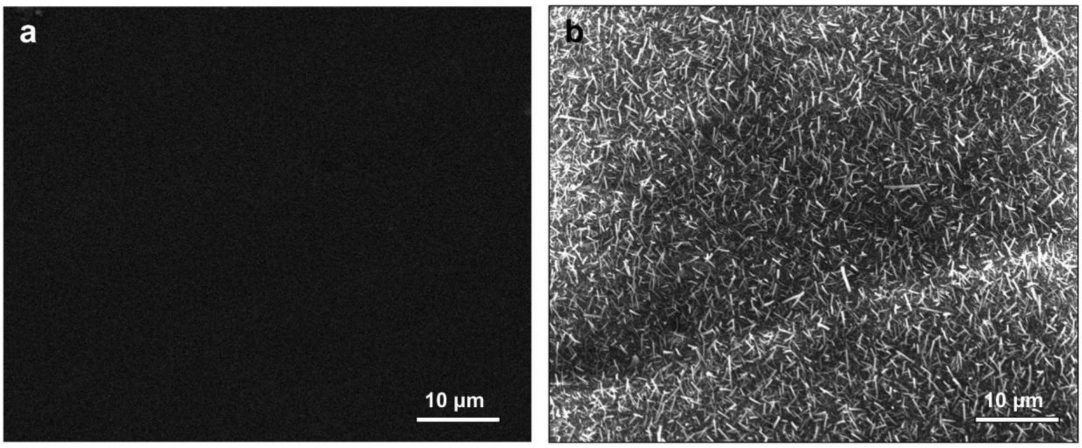

Figure S7. SEM images of **a**) BOPP film and **b**) the silica nanorod arrays grown on flexible substrate (plasma-treated BOPP film).

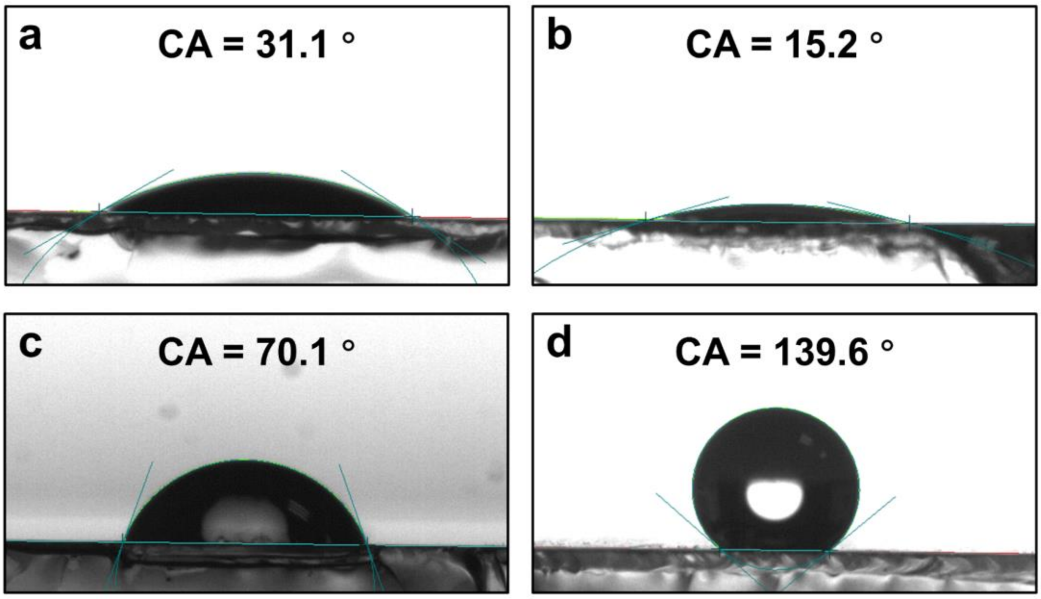

Figure S8. The contact angles of water on ITO based substrates. **a**) ITO glass, **b**) ITO glass with silica nanorod arrays, **c**) hydrophobically modified ITO glass, **d**) hydrophobically modified SiO_2 nanorod arrays on ITO glass with PFTS.

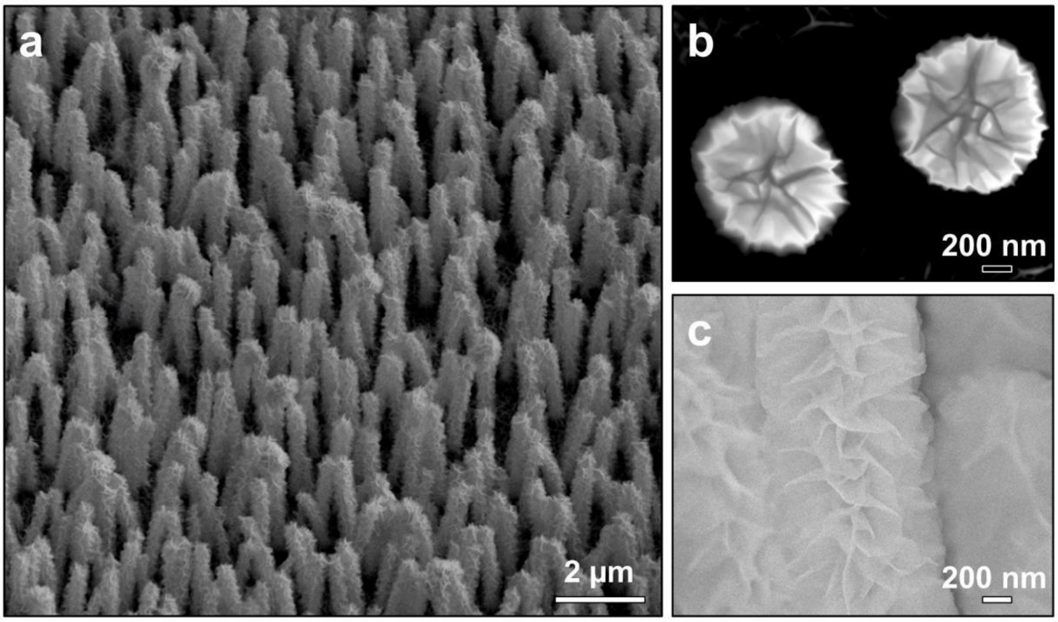

Figure S9. SEM images of $NiOOH@SiO_2$ arrays on ITO glass from **a**) oblique view, **b**) top view, and **c**) side view.

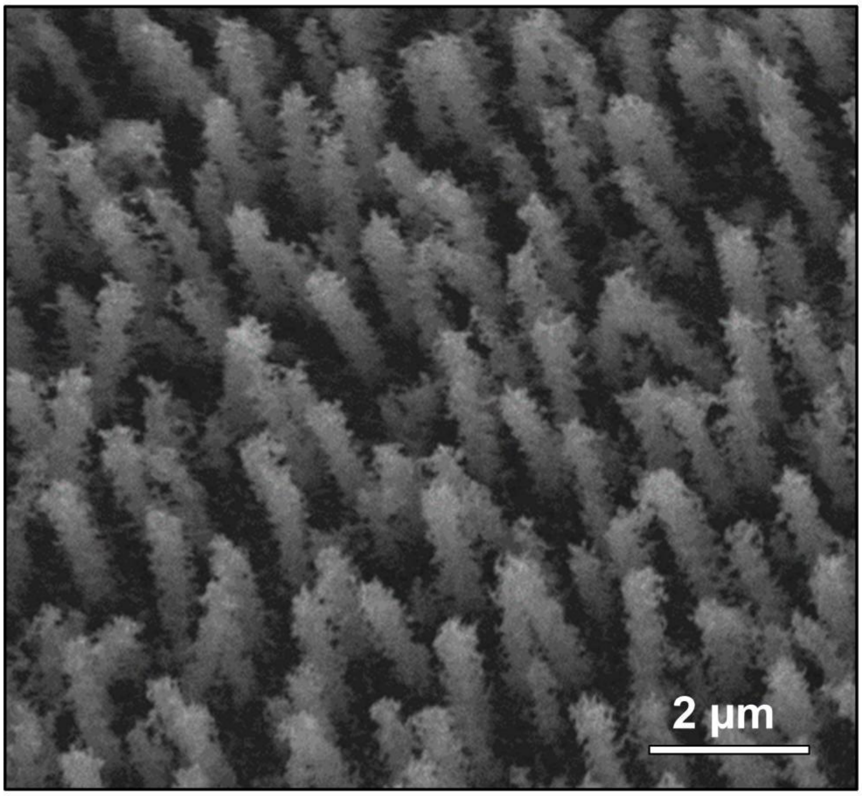

Figure S10. SEM image of Ni@SiO_2 nanorod arrays.

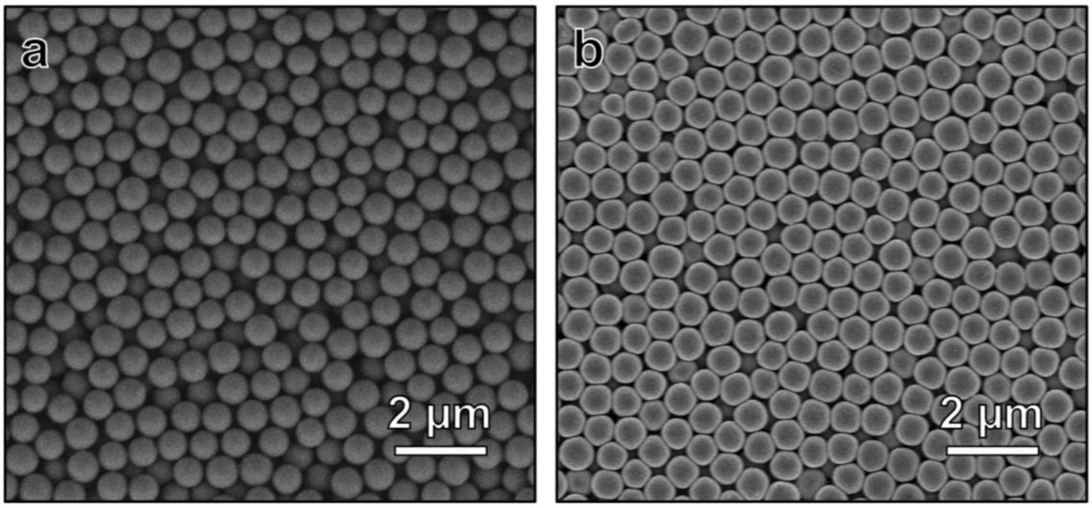

Figure S11. SEM images of **a**) silica spheres on glass, and **b**) Co@SiO_2-sphere. The 600-nm silica spheres were prepared by the Stöber method.

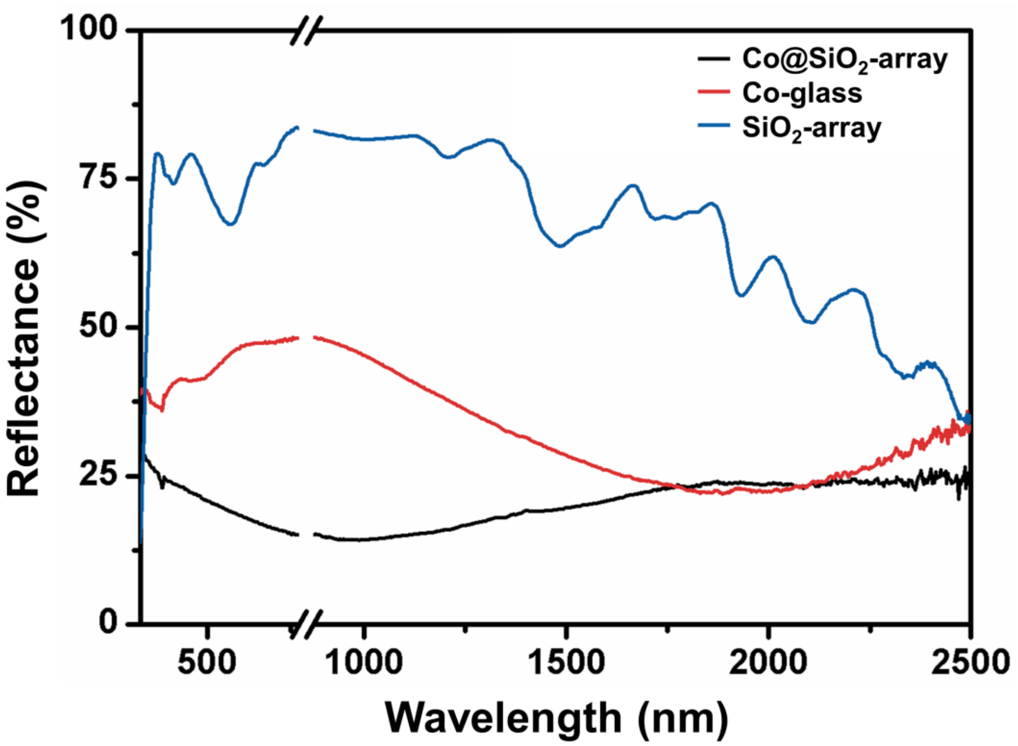

Figure S12. Diffuse reflectance spectra of Co@SiO_2-array, Co-glass, and SiO_2-array.

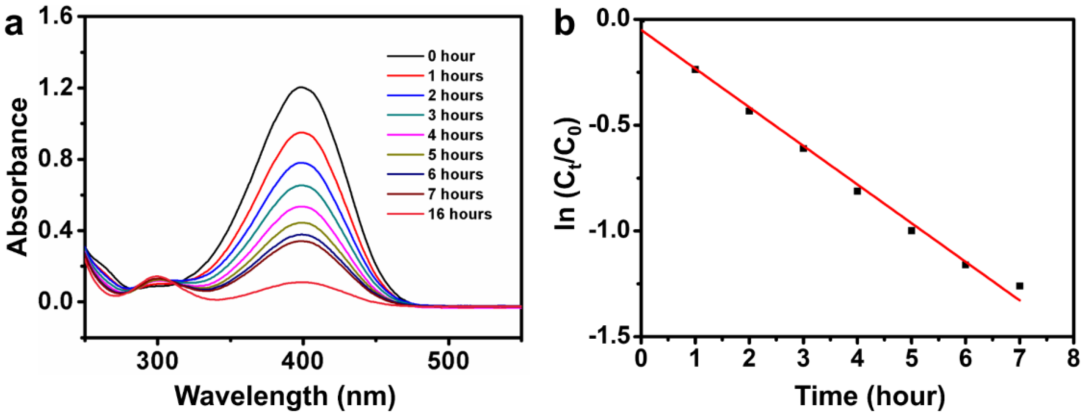

Figure S13. a) Time-dependent UV−vis spectra of the reaction solution in the presence of Au particles on glass in 1-hour intervals. **b**) Plot of ln (C_t/C_0) versus the duration of the reduction reaction.

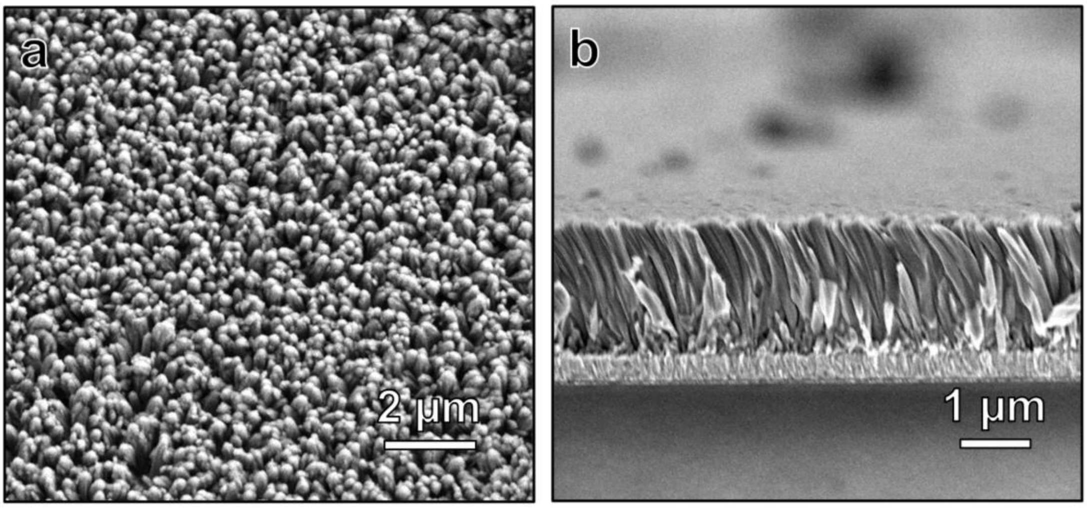

Figure S14. SEM images of TiO_2 nanorod arrays grown on the FTO glass slide from different viewing angles. The TiO_2 nanorod arrays were grown through the seedless SLS process in a hydrothermal autoclave. In a typical experiment, 0.5 g of PVP was dissolved in 5 mL of 1-pentanol by sonication under stirring, followed by the addition of 4.1 mL of deionized water, 100 µL of aqueous sodium citrate solution (0.2 M), 5 mL of concentrated hydrochloric acid (36.5% by weight) ,0.9 mL of saturated aqueous NaCl solution and 1 mL of ethanol. 230 µL of TBOT was then added to the above solution under constant stirring. After stirring for 3 minutes, the pre-treated FTO substrate was immersed in the above solution. The autoclave was heated at 150 ℃ for 6 hours in an electric oven. After removing the autoclave from the oven and cooling down to room temperature, the FTO glass slide was taken out, rinsed extensively with ethanol and then deionized water. Finally, the product was dried naturally.

Supplementary Table

Table S1. Comparison of catalytic activity.

Catalyst	Metal amount (mg)	Rate constant k (min^{-1})	Rate constant per unit mass k_{nor} ($min^{-1}\cdot mg^{-1}$)	Reference
Au@SiO_2 nanorod arrays-glass	$4.8*10^{-3}$	0.021	**4.2**	This work
Au-glass (control sample)	$4.8*10^{-3}$	0.003	**$6.2*10^{-1}$**	This work
AuPt@BGNs/Fe_3O_4	$9.2*10^{-3}$	8.6	$9.4*10^{2}$	1
Au@SiO_2	$3.2*10^{-1}$	$8.4*10^{-1}$	2.7	2
2% Au@2% Ag/ZIF-8	$1.4*10^{-1}$	$3.0*10^{-1}$	2.2	3
Fe_3O_4@SiO_2-Au@mSiO_2 microspheres	$2.1*10^{-2}$	$2.0*10^{-1}$	9.5	4
Au NPs/chitosan	$1.6*10^{-1}$	3.4	1.6*10	5

References

1. Gu, W.; Deng, X.; Jia, X.; Li, J.; Wang, E., *J. Mater. Chem. A* **2015**, *3*, 8793.
2. Lee, J.; Park, J. C.; Song, H., *Adv. Mater.* **2008**, *20*, 1523.
3. Jiang, H. L.; Akita, T.; Ishida, T.; Haruta, M.; Xu, Q., *J. Am. Chem. Soc.* **2011**, *133*, 1304.
4. Deng, Y.; Cai, Y.; Sun, Z.; Liu, J.; Liu, C.; Wei, J.; Li, W.; Liu, C.; Wang, Y.; Zhao, D., *J. Am. Chem. Soc.* **2010**, *132*, 8466.
5. Qiu, Y.; Ma, Z.; Hu, P., *J. Mater. Chem. A* **2014**, *2*, 13471.
